# Insulin Modulates the Immune Cell Phenotype in Pulmonary Allergic Inflammation and Increases Pulmonary Resistance in Diabetic Mice

**DOI:** 10.3389/fimmu.2020.00084

**Published:** 2020-02-11

**Authors:** Sabrina S. Ferreira, Maria A. Oliveira, Maristela Tsujita, Fernanda P. B. Nunes, Felipe B. Casagrande, Eliane Gomes, Momtchilo Russo, Wothan Tavares de Lima, Joilson O. Martins

**Affiliations:** ^1^Laboratory of Immunoendocrinology, Department of Clinical and Toxicological Analyses, School of Pharmaceutical Sciences, University São Paulo (FCF/USP), São Paulo, Brazil; ^2^Laboratory of Physiopathology of Experimental Lung Inflammation, Department of Pharmacology, Institute of Biomedical Sciences, University São Paulo (ICB/USP), São Paulo, Brazil; ^3^Laboratory of Hematology, Department of Clinical and Toxicological Analyses, School of Pharmaceutical Sciences, University São Paulo (FCF/USP), São Paulo, Brazil; ^4^Laboratory of Immunobiology, Department of Immunology, Institute of Biomedical Sciences, University São Paulo (ICB/USP), São Paulo, Brazil

**Keywords:** allergic inflammatory reaction, asthma, diabetes mellitus, insulin, immune cell phenotyping, eosinophils, respiratory mechanics, cytokines

## Abstract

**Introduction:** Reports have shown that the onset of diabetes mellitus (DM) in patients previously diagnosed with asthma decreases asthmatic symptoms, whereas insulin aggravates asthma. The present study evaluated the modulatory effect of insulin on the development of allergic airway inflammation in diabetic mice.

**Materials and Methods:** To evaluate the effects of relative insulin deficiency, an experimental model of diabetes was induced by a single dose of alloxan (50 mg/kg, i.v.). After 10 days, the mice were sensitized with ovalbumin [OVA, 20 μg and 2 mg of Al(OH)_3_, i.p.]. A booster immunization was performed 6 days after the first sensitization [20 μg of OVA and 2 mg of Al(OH)_3_, i.p.]. The OVA challenge (1 mg/mL) was performed by daily nebulization for 7 days. Diabetic animals were treated with multiple doses of neutral protamine Hagedorn (NPH) before each challenge with OVA. The following parameters were measured 24 h after the last challenge: (a) the levels of p38 MAP kinase, ERK 1/2 MAP kinases, JNK, STAT 3, and STAT 6 in lung homogenates; (b) the serum profiles of immunoglobulins IgE and IgG1; (c) the concentrations of cytokines (IL-4, IL-5, IL-10, IL-13, TNF-α, VEGF, TGF-β, and IFN-γ) in lung homogenates; (d) cells recovered from the bronchoalveolar lavage fluid (BALF); (e) the profiles of immune cells in the bone marrow, lung, thymus, and spleen; and (f) pulmonary mechanics using invasive (FlexiVent) and non-invasive (BUXCO) methods.

**Results:** Compared to non-diabetic OVA-challenged mice, OVA-challenged diabetic animals showed decreases in ERK 1 (2-fold), ERK 2 (7-fold), JNK (phosphor-54) (3-fold), JNK/SAPK (9-fold), STAT3 (4-fold), the levels of immunoglobulins, including IgE (1-fold) and IgG1 (3-fold), cytokines, including Th2 profile cytokines such as IL-4 (2-fold), IL-5 (2-fold), IL-13 (4-fold), TNF-α (2-fold), VEGF (2-fold), and TGF-β (2-fold), inflammatory infiltrates (14-fold), T cells, NK cells, B cells and eosinophils in the bone marrow, lung, thymus and spleen, and airway hyperreactivity. STAT6 was absent, and no eosinophilia was observed in BALF. Insulin treatment restored all parameters.

**Conclusion:** The data suggested that insulin modulates immune cell phenotypes and bronchial hyperresponsiveness in the development of allergic airway inflammation in diabetic mice.

## Introduction

Asthma affects ~1–18% of the population depending on the region. Global estimates show that these rates are increasing in all age groups, although these trends are notably increasing among children below 14 years of age (1). According to American Academy of Allergy Asthma and Immunology, asthma is a chronic inflammatory disease of the airways, and many factors can trigger an asthma attack. One type of asthma can be caused by allergens, such as pollen, moles and dust mites.

Severe Refractory Asthma (SRA) is the term used for severe asthma, and this condition afflicts only a small percentage of the asthma population (<5–10%) (2, 3). These patients suffer from poorly controlled symptoms and frequent exacerbations (4). Several environmental factors have roles in the pathogenesis of asthma, but multiple genes that confer disease susceptibility have also been described (5, 6), with evidence implicating genes related to respiration, particularly those linked to limitations of reversible or irreversible airflow (7).

Because of the extensive complexity of the pathophysiology of this disease, identifying its basis is difficult, which indicates that asthma should be further investigated. Data suggest that cells of the immune system are related to asthma, such as Th2 cells (8). Atopic individuals produce high levels of IgE in response to environmental allergens unlike non-atopic individuals, who synthesize other types of immunoglobulins, such as IgG and IgM, but little IgE (9, 10). This multicellular response is primarily characterized by eosinophil, neutrophil, CD4+ T lymphocyte, B lymphocyte, and mast cell activation, among other cells (9, 11–13). T lymphocytes and eosinophils are known to be critical in the development of asthma (6), which can be treated (7).

The association between eosinophils and airway hyperreactivity in asthma has been extensively investigated. Initial studies have revealed an important association between eosinophilic infiltrate and increased airway reactivity in both humans and different experimental models of asthma. Eosinophils release proteins that can damage the epithelial barrier, causing enzymatic degradation of mediators or impairing the bronchoprotective effect (14–16). Clinical (17, 18) and experimental data (19, 20) suggest that the immune response is impaired in type 1 diabetic individuals. Several aspects of this association have already been described: the onset of diabetes mellitus type 1 (DM1) in patients who have previously been diagnosed with asthma improves the asthmatic condition, but the treatment of diabetic patients with insulin, which is commonly used to treat DM1, aggravates asthma (21, 22). Inducing experimental diabetes in animals using chemicals, which selectively destroy pancreatic beta cells, is effective and simple (23). Alloxan is widely used to generate experimental models of DM1 (24, 25). Alloxan is a diabetogenic agent and has a selective toxic action on pancreatic beta cells, causing pancreatic islet necrosis. This agent is a glucose analog that binds to GLUT4 and accumulates in the beta cells of the pancreas, thus causing irreversible damage to pancreatic beta cells (26, 27).

Asthma and diabetes appear to have an antagonistic relationship (17, 21). In other studies, treatment with insulin in diabetic rats restored mast cell degranulation and histamine release as well as reactivity to ovalbumin (OVA). Relatively prolonged (12 days) treatment of animals with insulin resulted in gradual recovery of chemotaxis as evaluated *in vivo* (pleurisy, intravital microscopy) and *in vitro* (Boyden's chamber). Acute treatments with insulin (3 days) were ineffective (28). According to the results of a recent study of a model of late-phase pulmonary allergic inflammation, insulin restores mucus secretion and collagen deposition and increases cell migration, mainly eosinophils, in the airways of diabetic and asthmatic animals (29).

Further elucidation of the development of allergic airway inflammation in diabetic individuals is important. Because the role of insulin is not well-defined in chronic pulmonary allergic inflammation, the present study was designed to investigate the modulatory effect of insulin on the development of allergic airway inflammation in diabetic mice.

## Materials and Methods

### Animals

We used specific pathogen-free male BALB/c mice aged 8–12 weeks (20–25 g) at the beginning of the studies. The animals were maintained at 22°C under a 12 h light–dark cycle and were allowed access to food and water *ad libitum* throughout the observation period. This study was conducted in strict accordance with the principles and guidelines adopted by the Brazilian National Council for the Control of Animal Experimentation (CONCEA) and was approved by the Ethical Committee on Animal Use (CEUA) of the Faculty of Pharmaceutical Sciences (FCF) of University São Paulo (Permit Number: CEUA/FCF/490). All surgical procedures were performed under ketamine/xylazine anesthesia (270 and 30 mg/kg, respectively, s.c.), and all appropriate measures were employed to minimize suffering (Supplemental Figure 1).

### Assessment of the Levels of Signaling Molecules in Lung Tissues From Allergic Mice Using Immunoblotting

Protein concentrations were determined using a Pierce BCA Protein Assay Kit (Thermo Fisher Scientific Inc., Rockford, IL). Samples containing 20 μg of protein were separated using 10% sodium dodecyl sulfate-polyacrylamide gel electrophoresis and transferred to nitrocellulose membranes using the Amersham TE 70 PWR Semi-Dry Transfer system (Amersham Biosciences Corp., Piscataway, NJ, USA). For immunoblotting, nitrocellulose membranes were incubated in Tris-buffered saline-Tween (TBS-T) buffer (150 mM NaCl, 20 mM Tris, 1% Tween 20, pH 7.4) containing 5% non-fat dried milk for 1 h. Then, the membranes were washed with TBS-T buffer 3 times for 5 min each. Next, the membranes were incubated overnight with primary antibodies (1:1,000 dilution) against ERK 1/2 MAP kinases (Thr183/Tyr185), p38 MAP kinase (Thr180/Tyr182), JNK (phospho-54/SAPK-JNK), STAT-3 (124H6), and phospho-STAT 6 (Tyr641) diluted in 5% bovine serum albumin in TBST at 4°C. The antibodies were purchased from Cell Signaling Technology (Beverly, MA, USA). The membranes were incubated with anti-rabbit secondary antibody (1:10,000) for 1 h (Abcam) and developed using enhanced chemiluminescence detection of the nitrocellulose membrane. Band densities were determined by densitometry analysis using Image Studio Lite Version 5.2. The density of each band in each lane was normalized to the density of β-actin, which weighs 42 kDa and was a suitable choice of loading control for most of the proteins in the studied signaling pathway except for ERK 1/2 MAP kinases (42/44 kDa, respectively); therefore, glyceraldehyde 3-phosphate dehydrogenase (GAPDH), which weighs 36 kDa, was used for ERK 1/2 MAP kinases (Sigma Chemical Co., St. Louis, Mo, USA) (30).

### Determination of Serum Levels of Insulin, IgE, IgG1, and IgG2a

Blood samples were collected from the abdominal aorta of mice into a dry tube, and serum samples were stored at −70°C until determination of the levels of insulin, IgE, IgG1, and IgG2a according to the instructions of the Rat Insulin Enzyme ImmunoAssay Kit (SPI Bio, Massy Cedex, France), IgE Mouse Enzyme ImmunoAssay Kit (Abcam), IgG1 Mouse Enzyme ImmunoAssay Kit (Abcam), and IgG2a Mouse Enzyme ImmunoAssay kit (Abcam), respectively.

### Quantification of Cytokines in the Lung

After euthanasia of the animals, lobectomy of the left lobe of the lung was performed for cytokine analysis by enzyme-linked immunosorbent assays (ELISAs). The pulmonary lobe was macerated using 1 mL of RIPA buffer and then centrifuged, and the supernatant was collected for further analysis. The levels of cytokines (IL-4, IL-5, IL-10, IL-13, TNF-α, VEGF, TGF-β, and IFN-γ) were measured in lung homogenate supernatant samples by ELISA using commercial kits (R&D Systems, Inc., Minneapolis, MN, USA). Assays were performed according to the manufacturer's manual.

### Cells Recovered From the Bronchoalveolar Lavage Fluid

Mice were euthanized by a lethal dose of ketamine hydrochloride (360 mg/kg) and xylazine hydrochloride (36 mg/kg). The trachea was cannulated with polyethylene tubing (24 G3/4). The lungs were then lavaged by instillation of 1 mL of phosphate-buffered saline (PBS) (pH 7.4) three times for a total volume of 3 mL. The BALF was centrifuged at 1,500 rpm for 10 min, and the supernatant was frozen at −70°C until use in cytokine measurements. Pelleted cells were collected and resuspended in 1 mL of PBS. A single cell suspension was obtained after erythrocyte depletion (lysing solution, BD Biosciences), filtering and fixing for FACS. The total number of cells was quantified in a standard hemocytometer (Neubauer chamber; Herka, Berlin, Germany). Cytocentrifuge smears were stained using standard May-Grunwald and Giemsa solutions (Sigma Chemical Co). Differential cell counts were performed considering 100 cells per slide.

### Determination of Cellular Phenotypes in Different Organs and BALF

Flow cytometry was used to determine the percentage of cells that were positively labeled with antibodies [phycoerythrin (PE), fluorescein isothiocyanate (FITC), and allophycocyanine (APC) (eBioscience and ABCAM)] in different organs and BALF from the animals. The following staining parameters were employed: eosinophils were identified by SIGLEC-f (clone ESO-2440) and CD11b (clone M1/70), T cells were identified by CD3 (clone 17A2), CD45 (clone 30-F17), CD4 (clone RM4-4), CD8 (clone 53-6.7), TCR (clone UC7-13D5), B cell CD19 (Ebio1D3), and CD22 (clone ab25369), and NK cells (clone PK136). The isotype controls were mouse immunoglobulin IgG2b l FITC, rat IgG2a k PE, and mouse IgG1 k APC. Cells (10^6^ cells/mL) were labeled with 1 mg of monoclonal antibody and incubated for 20 min at room temperature (RT), and erythrocytes were lysed by adding 2 mL of 10% lysis solution (Lysing Solution, Becton Dickinson). Next, tubes were centrifuged at 1,500 rpm for 10 min, the supernatant was discarded, and the cell pellet was washed twice with PBS. After incubation, the cells were resuspended in 1% PFA/PBS and analyzed using a FACSCanto flow cytometer, and the data were analyzed with FlowJo software (Tree Star, Inc., USA). Positive controls and FMO were performed for each tissue evaluated.

### The Effect of Insulin on Airway Responsiveness

Bronchial hyperreactivity studies were divided into two groups. The first group was evaluated on 3 different days: the 2nd day of the challenge and the days of the 4th challenge and the 6th challenge (days of the experimental design: 29, 31, and 33, respectively). The second group was evaluated only on the 7th day of the challenge (day 36 of the experimental design). Bronchial hyperreactivity was assessed using the Penh (enhanced pause) values and the area under the curve (AUC) as the indexes. The baseline measurements were recorded and averaged for 3 min after acclimatization of the animals to the full body plethysmograph flow (FWBP) (Buxco Electronics Inc., Wilmington, NC, USA). The mice were exposed to saline (SAL) through a nebulizer for 2.5 min and then exposed to increasing concentrations of methacholine (MCh, Sigma-Aldrich, St. Louis, USA), which was also administered by nebulization (12 and 25 mg/mL) with an ultrasonic nebulizer. After each nebulization with MCh, data were recorded for 5 min. The results represent the area under the curve (AUC) obtained with increasing doses of MCh. The Penh values were calculated with an average of approximately 25 breaths, and the values were calculated and presented for each concentration (31).

### Study of Respiratory Mechanics

Twenty-four hours after the last challenge, the animals were anesthetized with ketamine (120 mg/kg, i.p.) and xylazine (12 mg/kg, i.p.) and remained in this condition throughout the procedure to evaluate respiratory mechanics. A tracheostomy was performed, and the jugular vein was cannulated for a subsequent injection of acetyl-β-methylcholine chloride (MCh, Sigma-Aldrich, St. Louis, MO). The mice were artificially ventilated (10 mL/kg tidal volume, 150 breaths/min, 3 cmH_2_O PEEP) (FlexiVent, SCIREQ, Quebec, Canada). Neuromuscular blockade was induced by an intraperitoneal injection of pancuronium bromide (1 mg/kg, Cristália, Brazil). The mice were exposed to PBS and increasing concentrations of MCh (0.03, 0.1, and 0.3 mg/kg). Assessments of respiratory mechanics were performed using a 3-s multifrequency volume perturbation. Raw (airway resistance) and Gtis (tissue viscance) were evaluated. The mean response after the PBS injection and the peak response after the injection of MCh at 0.03, 0.1, and 0.3 mg/kg were recorded (32).

### Statistical Analyses

Statistical analyses were performed using GraphPad 6 software (San Diego, CA, USA). Student's *t*-test and analysis of variance (ANOVA) followed by the Tukey–Kramer or Bonferroni test were used to perform comparisons. *P* < 0.05 were considered statistically significant.

## Results

### Effects of Diabetes and Insulin Treatment on the Levels of Signaling Molecules in Lung Tissues From Allergic Mice

**Figure 1:**
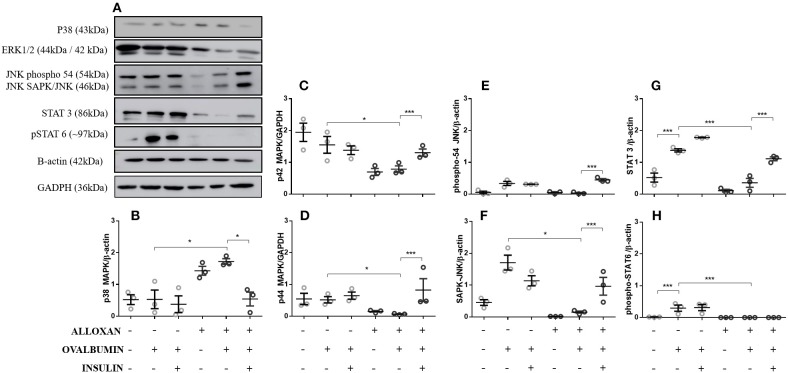
Levels of signaling molecules in lung homogenates. After collection of the lung, a lung tissue homogenate was created with RIPA buffer. **(A)** p38 MAP kinase (Thr180/Tyr182), ERK1/2 MAP kinase (Thr183/Tyr185), JNK (phospho-54/SAPK-JNK), STAT-3 (124H6), phospho-STAT 6 (Tyr641), β-actin protein and GAPDH levels in lungs from diabetic and non-diabetic mice subjected to an OVA challenge were determined by Western blotting. Relative **(B)** p38 MAP kinase; **(C)** ERK 1 (p42 MAPK); **(D)** ERK2 (p44 MAPK); **(E)** JNK (phosphor-54); **(F)** JNK (SAPK/JNK); **(G)** STAT3; and **(H)** Phospho-STAT6. Values represent the mean ± SEM from three independent experiments (*n* = 3 per group). ^*^p < 0.05; ^***^p < 0.001. The groups were tested with two-way analysis of variance followed by Tukey-Kramer *post-hoc* tests (GraphPad Prism version 6.0 for Windows, GraphPad Software, La Jolla, CA, USA).

Compared to the control group, in the lungs of non-diabetic allergic mice, the levels of JNK-phospho-54 (4-fold), JNK-SAPK/JNK (1.4-fold), STAT3 (2.6-fold), and pSTAT6 (11.5-fold) were significantly increased. In contrast, diabetic allergic mice showed significant reductions in ERK 1 (1.9-fold), ERK 2 (7.4-fold), JNK-phospho-54 (2.8-fold), JNK-SAPK/JNK (9.1-fold), STAT3 (3.8-fold), and total pSTAT6 levels and increased levels of p38 (4.5-fold) compared to non-diabetic allergic mice. Insulin treatment effectively restored the levels of ERK1/2, JNK, and STAT 3 in diabetic allergic mice but decreased the levels of p38 (4.4-fold) and did not affect pSTAT6 levels (Figure 1).

### Insulin Treatment Alters the Serum Levels of IgE, IgG1, and IgG2a in Diabetic Allergic Mice

**Figure 2:**
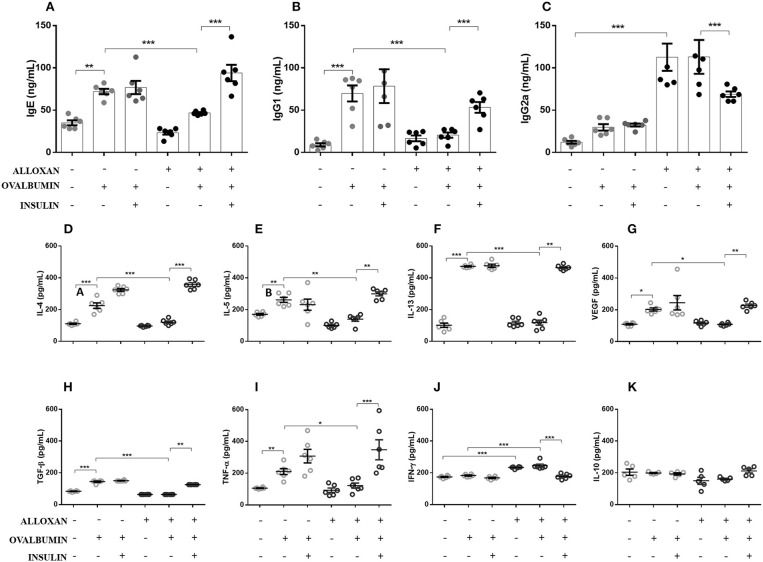
Effects of insulin on the secretion of immunoglobulins and cytokines in serum and lung homogenates, respectively. After collecting whole blood via cardiac puncture from animals in the different experimental groups, the serum was separated by centrifugation at 1,600 rpm for 20 min to evaluate the serum levels of **(A)** IgE, **(B)** IgG1, and **(C)** IgG2a. Lung tissues were collected and homogenized in RIPA buffer, and the supernatant was used in ELISAs to determine the levels of the following cytokines: **(D)** IL-4, **(E)** IL-5, **(F)** IL-13, **(G)** VEGF, **(H)** TGF-β, **(I)** TNF-α, **(J)** IFN-γ, and **(K)** IL-10. Values represent the mean±SEM from three independent experiments (*n* = 6 per group). ^*^p < 0.05; ^**^p < 0.01; ^***^p < 0.001. The groups were tested with two-way analysis of variance followed by Tukey–Kramer *post-hoc* tests (GraphPad Prism version 6.0 for Windows, GraphPad Software, La Jolla, CA, USA).

As shown in Figure 2, the serum of non-diabetic allergic mice showed significant increases in IgE (2-fold, Figure 2A), IgG1 (7-fold, Figure 2B) and IgG2a levels (2-fold, Figure 2C) compared to the control group. On the other hand, the serum of diabetic allergic mice exhibited decreased levels of IgE (1.5-fold, Figure 2A) and IgG1 (3.4-fold, Figure 2B) but increased levels of IgG2a (3.8-fold, Figure 2C). Insulin-treated mice exhibited increased serum levels of IgE (4-fold) and IgG1 (2.6-fold) but decreased IgG2a levels (1.6-fold) compared with untreated diabetic allergic mice (Figure 2).

### Effects of Diabetes on Cytokine Generation in the Lungs of Allergic Mice

As indicated in Figure 2, the lungs of non-diabetic allergic mice displayed increased concentrations of IL-4 (3.4-fold), IL-5 (1.5-fold), IL-13 (3.8-fold), VEGF (1.8-fold), TGF-β (1.7-fold), and TNF-α (2-fold). In contrast, the lungs of diabetic allergic mice showed significantly reduced levels of IL-4 (3.3-fold), IL-5 (1.8-fold), IL-13 (4-fold), VEGF (1.8-fold), TGF-β (2.2-fold), and TNF-α (2-fold) and increased levels of IFN-γ (1.3-fold). Treatment of diabetic mice with insulin restored the levels of the cytokines quantified except for IFN-γ, the levels of which were decreased by insulin treatment. IL-10 levels were not affected in any of the studied groups (Figures 2D–K).

### Effects of Insulin Treatment of the Number of Cells Recovered From the BALF of Diabetic Allergic Mice

**Figure 3:**
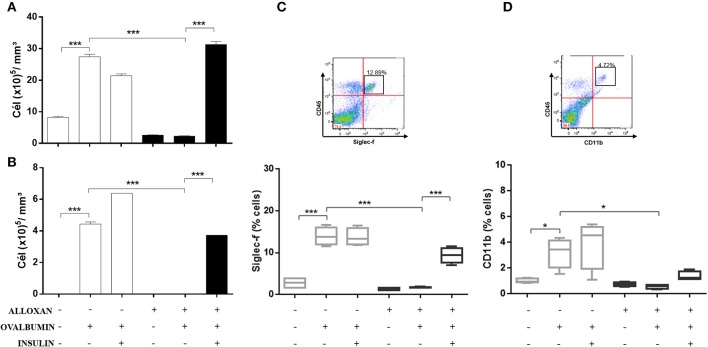
Characterization of leukocytes in BALF: the role of insulin. Animals were sensitized with OVA+Al(OH)_3_ (i.p.) 10 days after alloxan administration and 12 days after sensitization. Six days after the booster dose, the animals received 7 antigenic challenges daily with OVA or saline. NPH insulin treatment was performed between the 2 IU challenges of NPH insulin at 7 p.m. and 1 IU of NPH insulin at 7 h. Twenty-four hours after the last challenge, the animals were euthanized, and BALF was collected. **(A)** Total cells. **(B)** Eosinophils. **(C)** Siglec-f. **(D)** CD11b. Values represent the mean±SEM from three independent experiments. ^*^p < 0.05; ^***^p < 0.001. The groups were tested with two-way analysis of variance followed by Tukey–Kramer *post-hoc* tests (GraphPad Prism version 6.0 for Windows, GraphPad Software, La Jolla, CA, USA).

Figure 3 shows significant increases in the numbers of total cells (3.6-fold, Figure 3A) and eosinophils (4.2-fold, Figure 3B) in the BALF of OVA-challenged non-diabetic mice. In contrast, the BALF of diabetic allergic mice showed marked reductions in the numbers of total cells (11.7-fold) and eosinophils (4.43-fold). Insulin treatment of diabetic OVA-challenged animals restored total cellularity and eosinophilic migration compared to the group that did not receive insulin treatment. The immunophenotypic analysis revealed increases in the populations of Siglec-f-positive (5-fold) (Figure 3C) and CD11b-positive (3.2-fold) cells (Figure 3D) after the OVA challenge. The diabetic OVA challenge reduced CD11b-positive (5.3-fold) and Siglec-f-positive (8.2-fold) cells. Insulin treatment restored the migration of these populations.

### Effects of Insulin on Cellular Phenotypes in Selected Organs of Allergic Mice

**Figure 4:**
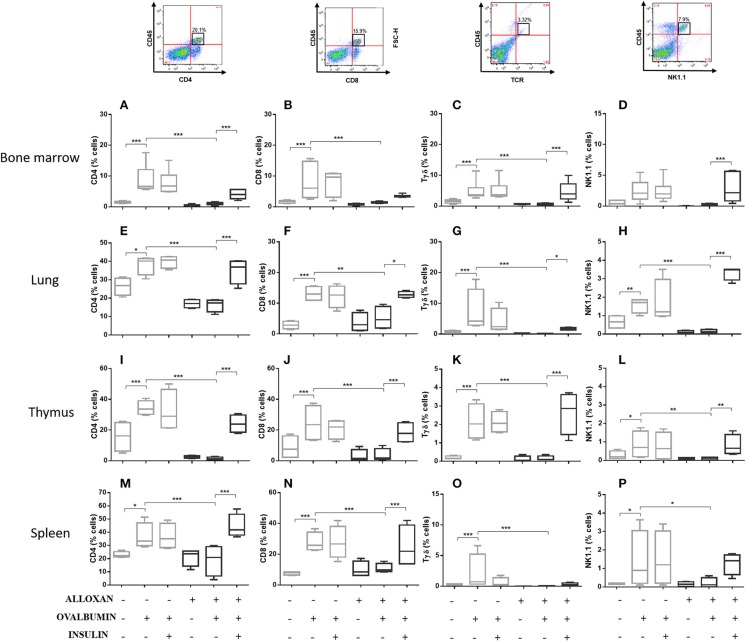
Profile analysis of recruited T lymphocytes and NK cells. Acquisitions were performed using an FACSCanto flow cytometer, and the data were analyzed using FlowJo software (Tree Star, Inc., USA). We analyzed 20,000 events. Representative dot plots showing the gating strategy used to identify T cells and NK cells in the bone marrow, **(A)** CD4, **(B)** CD8, **(C)** Tγδ, and **(D)** NK1.1; lung, **(E)** CD4, **(F)** CD8, **(G)** Tγδ, and **(H)** NK1.1; thymus, **(I)** CD4, **(J)** CD8, **(K)** Tγδ, and **(L)** NK1.1; and spleen, **(M)** CD4, **(N)** CD8, **(O)** Tγδ, and **(P)** NK1.1. Values represent the mean ± SEM from three independent experiments. ^*^p < 0.05; ^**^p < 0.01; ^***^p < 0.001. The groups were tested with two-way analysis of variance followed by Tukey–Kramer *post-hoc* tests (GraphPad Prism version 6.0 for Windows, GraphPad Software, La Jolla, CA, USA).

**Figure 5:**
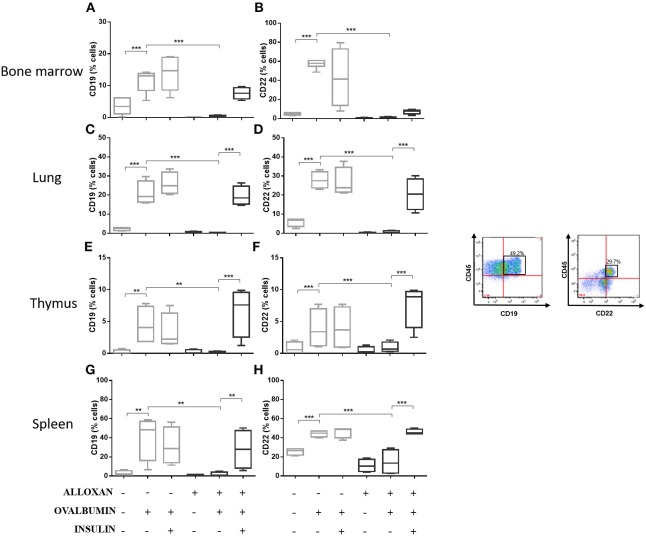
Profile analysis of recruited B lymphocytes. Acquisitions were performed using an FACSCanto flow cytometer, and the data were analyzed using FlowJo software (Tree Star, Inc., USA). We analyzed 20,000 events. Representative dot plots showing the gating strategy used to identify B cells in the bone marrow, **(A)** CD19 and **(B)** CD22; lung, **(C)** CD19 and **(D)** CD22; thymus, **(E)** CD19 and **(F)** CD22; and spleen, **(G)** CD19 and **(H)** CD22. Values represent the mean ± SEM from three independent experiments (*n* = 3 per group). ^**^p < 0.01; ^***^p < 0.001. The groups were tested with two-way analysis of variance followed by Tukey–Kramer *post-hoc* tests (GraphPad Prism version 6.0 for Windows, GraphPad Software, La Jolla, CA, USA).

**Figure 6:**
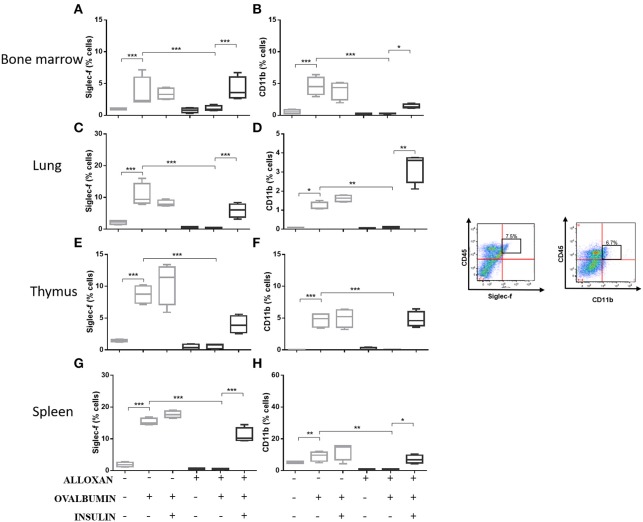
Profile analysis of recruited eosinophils. Acquisitions were performed using an FACSCanto flow cytometer, and the data were analyzed using FlowJo software (Tree Star, Inc., USA). We analyzed 20,000 events. Representative dot plots showing the gating strategy used to identify eosinophils in the bone marrow, **(A)** Siglec-f and **(B)** CD11b; lung, **(C)** Siglec-f and **(D)** CD11b; thymus, **(E)** Siglec-f and **(F)** CD11b; and spleen, **(G)** Siglec-f and **(H)** CD11b. Values represent the mean ± SEM from three independent experiments (*n* = 3 per group). ^*^p < 0.05; ^**^p < 0.01; ^***^p < 0.001. The groups were tested with two-way analysis of variance followed by Tukey–Kramer *post-hoc* tests (GraphPad Prism version 6.0 for Windows, GraphPad Software, La Jolla, CA, USA).

The immunophenotypic analysis of cells in the bone marrow, lung, thymus and spleen revealed similar phenotype profiles. Among T cell populations and NK cells, the bone marrow of non-diabetic allergic mice showed significant increases in the populations of CD4-positive (6-fold), CD8-positive (4.8-fold), and Tγδ-positive (3.3-fold) T cells and NK1.1-positive (5.5-fold) NK cells (Figures 4A–D). Lung tissues from this group of mice displayed increased numbers of CD4-positive (1.4-fold), CD8-positive (4.6-fold), Tγδ-positive (10.7-fold), and NK1.1-positive (2.3-fold) cells (Figures 4E–H). In the thymus, we observed increased populations of CD4-positive (2.1-fold), CD8-positive (2.8-fold), Tγδ-positive (10.7-fold), and NK1.1-positive (3.3-fold) cells (Figures 4I–L). We also observed increased populations of CD4-positive (1.5-fold), CD8-positive (3.6-fold), Tγδ-positive (7.4-fold), and NK1.1-positive (7-fold) cells in the spleen (Figures 4M–P). Increases in the proportions of CD19-positive (5.2-fold) and CD22-positive B cells (12.1-fold) were observed in the bone marrow (Figures 5A,B), increases in the proportions of CD19-positive (9.1-fold) and CD22-positive B cells (4.7-fold) were observed in the lung (Figures 5C,D), increases in the proportions of CD19-positive (21-fold) and CD22-positive B cells (4.3-fold) were observed in the thymus (Figures 5E,F), and increases in the proportions of CD19-positive (14.4-fold) and CD22-positive B cells (1.7-fold) were observed in the spleen (Figures 5G,H). Increases in the Siglec-f-positive and CD11b-positive populations were observed in the bone marrow, lung, thymus, and spleen, which exhibited 3.5- and 9.6-fold (Figures 6A,B), 4.8- and 2.9-fold (Figures 6C,D), 6.6- and 5-fold (Figures 6E,F), and 8- and 1.6-fold (Figures 6G,H) increases, respectively. Diabetic animals treated with OVA showed a significant reduction in the immune cell profiles. Insulin treatment restored the cell profiles determined by flow cytometry (also see Supplemental Table 1 and Supplemental Figures 2, 3).

### Effects of Multiple Challenges on the Respiratory Function of Diabetic Allergic Mice Assessed Using BUXCO

**Figure 7:**
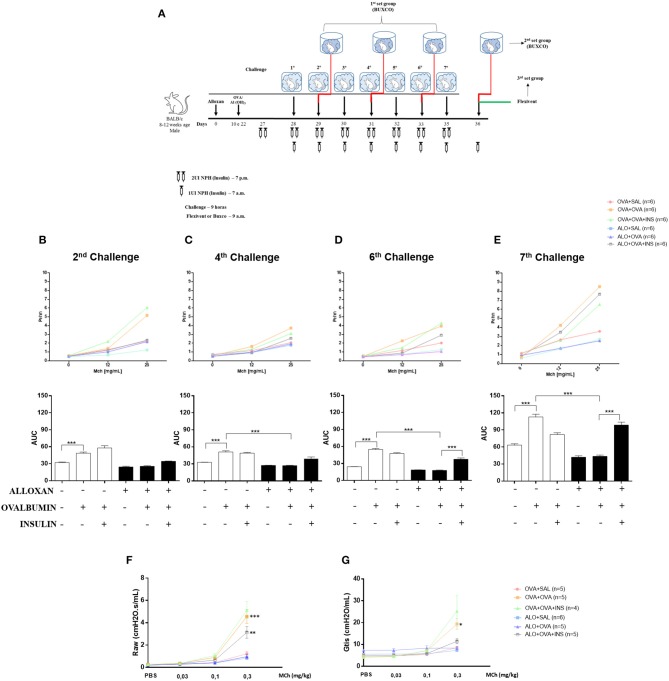
The role of insulin in respiratory mechanics. Methacholine capacity (MCh) was evaluated in mice. **(A)** Experimental protocols for BUXCO and FlexiVent. **(B)** The 2nd challenge. **(C)** The 4th challenge. **(D)** The 6th challenge. **(E)** The 7th challenge. **(F)** Raw. **(G)** Gtis. Values represent the mean±SEM from three independent experiments (*n* = 4–6 animals per group). ^*^p < 0.05; ^**^p < 0.01; ^***^p < 0.001. The groups were tested with two-way analysis of variance followed by Tukey–Kramer *post-hoc* tests for the BUXCO condition and Bonferroni tests for the FlexiVent condition (GraphPad Prism version 6.0 for Windows, GraphPad Software, La Jolla, CA, USA).

After 2 challenges with OVA, non-diabetic mice presented an increase (1.5-fold) in airway responsiveness compared to the SAL-challenged mice. Induction of diabetes did not change this pattern, and diabetic animals could not respond to the OVA challenge, resulting in no changes in lung responsiveness. The initiation of insulin treatment induced a slight increase in bronchial hyperreactivity, but the difference was not significant (Figure 7B).

After 4 OVA challenges, airway responsiveness was similar to the pattern observed after the second challenge (Figure 7C).

After 6 challenges, insulin treatment significantly increased the hyperresponsiveness of diabetic OVA-challenged animals to methacholine (2.2-fold) (Figure 7D).

After 7 challenges, non-diabetic OVA-challenged mice showed increased airway responsiveness (1.8-fold) compared to the SAL-challenged mice. The induction of diabetes did not alter basal airway responsiveness compared to non-diabetic SAL-challenged animals, and diabetic animals were unable to respond to the AO challenge, showing unchanged airway responsiveness. Insulin treatment restored airway responsiveness (Figure 7E).

### Effects of Insulin on Airway Resistance and Tissue Viscance

The respiratory mechanics of the animals were measured 24 h after the last challenge (7 challenges). Non-diabetic OVA-challenged mice exhibited increased Raw (2.2-fold) and Gtis (4-fold) compared to the SAL-challenged mice. The induction of diabetes did not alter these parameters compared to those of non-diabetic animals. SAL-challenged and diabetic animals were unable to respond to the AO challenge. Allergic diabetic mice treated with insulin exhibited significant increases in Raw (Figure 7F) and Gtis (Figure 7G) to values similar to those observed in allergic non-diabetic mice.

## Discussion

The results presented in this study suggest that insulin modulates the development of the late pulmonary allergic inflammatory reaction in a diabetic murine model due to its ability to restore (a) ERK 1/2 MAP kinases, JNK, and STAT 3 in the lung; (b) the profile of immunoglobulins present in the serum (IgG1 and IgE); (c) the concentrations of cytokines (IL-4, IL-5, IL-13, TNF-α, VEGF, and TGF-β) in lung homogenates; (d) cell migration in BALF; (e) the profile of immune cells (CD4, CD8, Tγδ, NK1.1, CD19, CD22, SIGLE-f, and CD11b) in the bone marrow, lung, thymus, and spleen; and (f) airway hyperreactivity.

Insulin was discovered in the 20's, much research is still needed to unravel its role in the body. The results shown here suggest strongly believe that important undermined details were highlighted. First of all, many physicians focus on controlling hyperglycemia in diabetes, while insulin resistance and lack of insulin production remain untouched. In this asthma model we suggest that in diabetic mice, previously challenged with OVA, reduced cytokines production and defective expression of adhesion molecules, may partially explain defective neutrophils migration and ineffective inflammation (33). More recently we have also shown that insulin treatment might be involved with airway remodeling since we obtained better collagen airway deposition and mucus secretion when diabetic mice were insulin treated (29). Our group also studied that lack of insulin may cause permanent defect in bone marrow compartment (34), since bone marrow derived macrophages from diabetic rats differ phenotypically from control, healthy animals. We rescue this previous study adding asthma to the system. Accordingly to American academy of allergy asthma and immunology, asthma patients are also more favorable to develop autoimmune diseases, therefore, in these cases, bone marrow cells are strongly required to be delivered in the blood stream as different sources of immune and inflammatory cells. Clinical asthma appears to be less severe when existing diabetes mellitus. Data from epidemiological studies in children with type-1 diabetes mellitus (17, 35) are consistent with data from previously published studies (21, 36), suggesting an inverse relationship between atopy and diabetes mellitus. It is possible to speculate that asthma is suppressed in type I diabetic individuals because there is a relative lack of insulin which, in turn, would allow asthma to manifest itself clinically.

Insulin also regulates the metabolic activity, gene transcription and growth of several cell types by modulating the activity of several proteins involved in intracellular signaling during the development of the inflammatory response (37, 38). Proteins of the MAPK pathway have important roles in the activation and differentiation of T cells (39, 40). Studies have suggested that enhanced phosphorylation of ERK may be related to polarization of T cells to Th2 cells (40), while phosphorylation of p38 favors polarization to the Th1 profile (41–43). In 2007, Viardot et al. (44) showed that insulin promotes the differentiation of cells into Th2 cells, reducing the proportion of Th1 cells and thus reducing the IFN-γ/IL-4 ratio. These findings were related to increased phosphorylation of ERK and reduced p-p38. We analyzed the expression of other molecules important for the development of allergic airway inflammation, such as STAT3 (45), pSTAT6 (46), and JNK (40). We observed an increase in the expression of all these molecules in macerated lung samples from non-diabetic animals challenged with OVA. The induction of diabetes reduced the expression of these molecules, and insulin re-established their expression levels. We found similar results in our models for cytokines and immunoglobulin Th2 and Th1 profiles. A negative correlation between asthma and diabetes, which is similar to the correlation between Th1 and Th2 responses, has been suggested (47–49).

An increase in TGF-β levels is an early event in remodeling that precedes measurable morphological changes. In sensitized and challenged animals, TGF-β expression increases a few hours after stimulation and reaches the maximum level after 7 days depending on the stimulus (50). VEGF, the levels of which are increased in patients with asthma (51), is a mediator of vascular and extravascular remodeling and inflammation that increases antigen sensitization and is required for adaptive Th2 inflammation (52). These two indicators suggest that our model represents late pulmonary allergic inflammation because after the OVA challenges, the animals showed increases in TGF-β and VEGF. Inhaled TNF-α increases bronchial responsiveness to methacholine in normal individuals and patients with asthma (53).

Bendelac et al. demonstrated that several lymphocyte populations are related to the pulmonary allergic inflammatory response. To characterize the expression of surface molecules during the onset and development of the asthmatic inflammatory response, we studied several T lymphocytes (CD4+, CD8+, and Tγδ), NK cells, B lymphocytes (CD22 and CD19) and eosinophils (Siglec-f and CD11b) in different animal tissues. The CD4+ T lymphocyte population showed an increase in all the studied organs. Standard Th2 CD4+ T cells are required for the allergic response in the lung, and elimination of this population ameliorates the asthmatic phenotype (54, 55). In addition, Gavett et al. (54) demonstrated that CD4+ T lymphocytes can mediate surface membrane hyperreactivity, as well as increased numbers of lymphocytes and eosinophils in BALF, in mice subjected to an antigen challenge compared to control mice. In addition, Hamelmann et al. (56) showed that CD8+ T cells mediate the increase in IL-5 levels, leading to eosinophil infiltration and airway hyperreactivity. These NK lymphocytes were shown to function synergistically with Tαβ lymphocytes in the pathogenesis of allergic asthma as animals deficient in these cells display reduced pulmonary eosinophilia, airway hyperreactivity and a decreased concentration of Th2-type cytokines compared to controls (57). Similar results were obtained for mice deficient in Tγδ cells, which also showed decreased pulmonary eosinophilia in addition to reduced levels of IgG1, IgE, and IL-5. Evidence from animal models strongly indicates the presence of NK cells in the development of allergic diseases of the airways as these cells express a T-cell invariant receptor (Tγδ) in mice (Vα14) and in humans (Vα24). Activation of invariant Tγδ-positive NK cells leads to the rapid production of a range of inflammatory cytokines, including IL-4. Studies have shown that mice deficient in NK cells present decreased eosinophilia and thus decreased bronchial hyperresponsiveness (58). In addition, they also show a decrease in the concentration of Th2-type cytokines compared to controls (59). A decrease in pulmonary eosinophilia was also observed in mice deficient in Tγδ in addition to reduced concentrations of IgG1, IgE and IL-5 (60). In addition, the proportion of Tγδ cells in the BALF of asthmatic patients is higher than that in non-asthmatic patients (61). Studies in the literature suggest that Tγδ and NK cells can regulate the differentiation of the Th1 and Th2 responses (62) and thus modulate the development of allergic lung diseases. Among the populations of CD4+ T cells, the Th2 phenotype represents a fundamental population because elimination of this subpopulation by antibodies reduces bronchial hyperreactivity and pulmonary eosinophilic infiltration (53, 63). Several studies have shown a crucial role of T lymphocytes and the production of Th2 cytokines in the development of asthma (14, 29, 57, 63). However, the role of B cells in allergic asthma remains undefined, with the exception of the well-known ability of B lymphocytes to produce IgE-specific immunoglobulin after Th2 cell signaling (10, 11). To further clarify the role of B lymphocytes in asthma, Voogth and colleagues (64) revealed a crucial role for B lymphocytes in the development of asthma in rat models. The transfer of sensitized B lymphocytes to naive animals resulted in increases in hyperreactivity and inflammation of the airways after an antigen challenge. These findings suggested that B lymphocytes can induce an asthmatic response without the help of T lymphocytes. These data provide new insight into the mechanisms by which B cells promote asthma and suggest that an increase in this population is linked not only to increased serum IgE in animals but also to hyperreactivity and inflammation of the airways. We found that the induction of diabetes reduced the lymphocyte population. Diabetic animals did not respond to the OVA challenge. Insulin treatment restored the different lymphocyte populations in the various organs studied, suggesting that insulin at adequate concentrations seems to be required for normal cell migration during the course of the inflammatory process (29, 33). These results may also suggest that there is a relationship between insulin levels and the affected function and numbers of the innate immune cell, although studies show involvement of immunological system to the pathophysiological development of metabolic abnormalities, mechanisms linking immunity to metabolic disfunctions such as the ones involving variations of levels of insulin are still needed to be elucidated (65). Our results show increased T cells in the bone marrow of diabetic allergic animals following insulin treatment, suggesting a correlation with a possible effect on the number and function of innate lymphoid cells.

Given that all parameters studied lead to increased lung responsiveness, we evaluated the mechanics of the lung in two different conditions: without anesthesia (BUXCO) and under anesthesia (FlexiVent). We found that bronchial responsiveness did not change with the use or absence of anesthesia. To evaluate the effects of the antigenic challenges in the airways and insulin treatment, we assessed insulin groups after 2, 4, and 6 challenges. Another group was evaluated 24 h after the last challenge to assess the cumulative effect of all antigenic challenges in the airways and insulin treatment. Animals that were challenged only once with methacholine had greater bronchial responsiveness than animals that received this challenge three times. Notably, the normal inflammatory process seems to depend on the availability of insulin (33), and an impaired response to OVA in diabetic subjects may be primarily linked to an ongoing insulin deficiency rather than secondary hyperglycemia (29, 33). In fact, insulin induced allergic airway inflammation and association between insulin and pulmonary function worsening is still controversial. Honiden and Gong (66) has suggested that insulin therapy in ICU (intensive care unit) patients prevent development of acute lung injury. However, we understand that this particular situation may not embrace mice model used in our study. There are several studies suggesting that insulin modulates pulmonary allergic inflammation at all stages. Cavalher-Machado et al. (67) showed that mast cell degranulation is modulated by insulin in rats early in the antigen challenge. These results help to understand the mechanisms of negative correlation of asthma in a diabetic individual. It is important to emphasize the importance of the mast cell in the pathophysiology of asthma, due to its secretory activities that act on the initial response in allergic patients (68) and animal models (69). It is known that inhaled insulin therapy has been a failure over the years (70). Olek et al. suggests that insulin may dec1line pulmonary functions in disease such as asthma or chronic obstructive pulmonary disease, but raises that there is still need long term studies to evaluate this information. Cavaiola and Steven (71) described some years before that no differences regarding pulmonary function decrease were shown between patients that inhaled insulin and placebo (71). In fact, there is still no clear understanding of insulin interference in lungs air flow. Recently, insulin resistance has been related to asthma like symptoms and this might enlighten our knowledge about insulin effect in normal individuals compared to those who develop diabetes. Indeed, since it is not possible to inject insulin in healthy patients (since they can die with hypoglycemia), insulin interference in healthy lungs remains still unraveled. In here, the treatment with multiple doses of insulin restored the parameters involved in the late inflammatory response, even without normalizing glycemia. These findings suggest that insulin plays an important modulatory role in the development of allergic airway inflammation in diabetic mice. Although the data presented here and in the literature suggest that asthma symptoms are attenuated by DM1, the related mechanisms are not fully understood, leading us to believe that more studies are needed since other mechanisms may be involved in this negative correlation. CTLs may attack insulin-producing cells, and activation of CTLs requires type I cytokines, which would decrease Th2-type immune responses. Thus, asthma resistance in diabetic patients may be due to immune system dysfunction, which also causes diabetes. Therefore, more in-depth studies are required in our future work.

The results presented here provide important information about the modulatory effect of insulin on the allergic inflammatory response induced experimentally by OVA in diabetic mice.

## Data Availability Statement

All datasets generated for this study are included in the article/Supplementary Material.

## Ethics Statement

This study was conducted in strict accordance with the principles and guidelines adopted by the Brazilian National Council for the Control of Animal Experimentation (CONCEA) and approved by the Ethical Committee on Animal Use (CEUA) of the Faculty of Pharmaceutical Sciences (FCF) of University São Paulo (Permit Number: CEUA/FCF/490).

## Author Contributions

SF and JM conceived and designed the experiments. SF, MO, MT, FN, FC, and EG performed the experiments. SF, MR, WT, and JM analyzed the data. MR, WT, and JM contributed reagents, materials, and analysis tools. SF and JM wrote the paper with the assistance of all the authors.

### Conflict of Interest

The authors declare that the research was conducted in the absence of any commercial or financial relationships that could be construed as a potential conflict of interest.
