# Supplementary material for: Insulin Modulates the Immune Cell Phenotype in Pulmonary Allergic Inflammation and Increases Pulmonary Resistance in Diabetic Mice
Source: Front Immunol. 2020 Feb 11;11:84. doi: 10.3389/fimmu.2020.00084 (PMC7026190; doi:10.3389/fimmu.2020.00084)
Supplement: Supplementary file 1 [file Data_Sheet_1.pdf]

# SUPPLEMENTAL DATA

## Supplemental Materials and methods

### Induction of Diabetes Mellitus

Diabetes mellitus was induced by intravenous injection of alloxan monohydrate (50 mg/kg; Sigma Chemical Co., St. Louis, MO, USA) dissolved in physiologic saline (SAL, 0.9% NaCl). Control mice were injected with physiologic SAL only. After 10 days, the presence of diabetes was verified by blood glucose concentrations higher than 300 mg/dL, which were determined with a blood glucose monitor (Accu-Chek Advantage II, Roche Diagnostica, São Paulo, São Paulo, Brazil), in blood samples obtained from mouse tails[1,2]

### Induction of Allergic Asthma

Mice were sensitized on days 10 and 22 by intraperitoneal (i.p.) injection containing 20 µg of OVA (Sigma, USA) and 2 mg of aluminum hydroxide [Al(OH3); Reheis Inc., USA] in PBS to a total volume of 0.2 mL. Sensitized and control mice were challenged by multiple exposures to aerosol (5% OVA in PBS) from an ultrasonic nebulizer (ICEL US-800, São Paulo, Brazil), delivering particles of 0.5–10 µm diameter at approximately 0.75 cc/min for 30 min. Challenges were performed daily for 7 days (28–33 and 35). The experiments were performed 24 h after the last challenge[1,3].

### Insulin treatment

Diabetic mice received 2 IU and 1 IU of insulin subcutaneously 14 h before the OVA challenges (07:00 p.m.) and half doses (07:00 a.m.) of insulin 2 h before each of the 7 OVA challenges (Figure 1), and control mice received 1 IU of insulin 2 h before each of

the alloxan (AO) challenges. Analyses were performed 2 h after the last dose of insulin[1,4].

**Determination of cellular phenotypes in different organs and BALF**

Regarding the immunophenotypic profile, to identify and analyze the expression of surface molecules in lung, spleen, thymus and bone marrow cell population a dot plot scatter plot was made in which the abscissa axis corresponds to the FSC parameter (cell size) and the ordinate axis to the SSC parameter (cellular complexity). A region around the total cells was delimited, excluding the debris. The percentage of positive cells was evaluated in the upper right quadrant, corresponding to double positive populations for the markers.

**Lung, spleen and thymus cellularity**

Lung tissue was aseptically removed and digested using collagenase IV (2 mg/mL) and DNase I (1 mg/mL) (Sigma-Aldrich) at 37°C for 30 min, after the preincubation for the lungs, and the same protocol was used for all organs. A single cell suspension was obtained after maceration (Potter-Elvehjem and glass tube, Sigma-Aldrich), erythrocyte depletion (lysing solution, BD Biosciences), filtration and fixing (2% PFA). Total cell counts were determined as described.

**Bone marrow cellularity**

For the cell preparations, femurs were removed aseptically. Bone marrow cells were flushed from these cells using sterile RPMI 1640 (Gibco, Thermo Fisher) supplemented with 10% fetal bovine serum (Invitrogen), and flow cytometry was used. Total cell counts were determined as described.

**Statistical analyses**

Statistical analyses were performed using GraphPad 6 software (San Diego, CA, USA), Student's *t*-test and analysis of variance (ANOVA) followed by the Tukey-Kramer test were used to perform comparisons. A p value lower than 0.05 was considered statistically significant.

## Supplemental figure 1

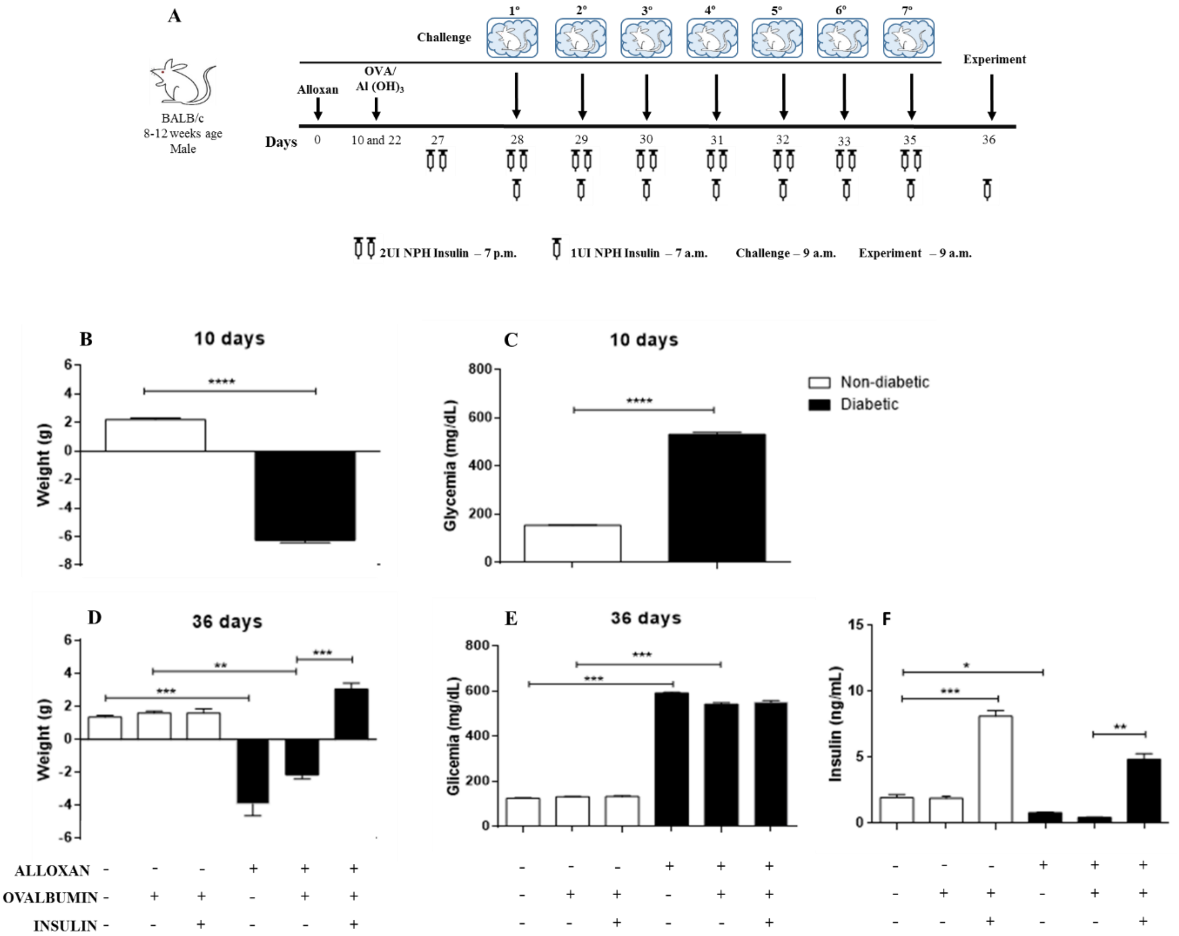


**Supplementary Figure 1. Experimental protocols and General characteristics of the diabetic mice**. **A)** Mice were rendered diabetic by injection of alloxan (50 mg/kg, i.v.). Ten days later, they were sensitized 2X intraperitoneally with OVA and Al(OH$^3$) and then subjected to aerosol antigen challenges (7 days) with OVA (1%, w/v in PBS) or PBS alone. Diabetic mice received 2 and 1 IU of insulin subcutaneously 12 h before the OVA challenges (07:00 p.m.) and half doses (07:00 a.m.) of insulin 2 h before each of the 7 OA challenges; 24 h after the last challenge, serum, lungs, bone marrow, thymus, spleen, and BALF were collected for further analysis. Nondiabetic mice received 1 IU insulin subcutaneously 2 h before the OVA challenges. **B**) The weights of the animals belonging to the nondiabetic and diabetic groups were evaluated 10 days after the injection of alloxan (50 mg/kg) or saline. **C)** Glycemia was determined by glucose monitoring (Advantage, Lilly) 10 days after the intravenous injection of alloxan (50 mg/kg) using blood samples obtained from the tail end. During the experiments, only animals with glycemia over 300 mg/dL were used. **D)** The weight was again evaluated on day 36 of the experiment. **E)** Glycemia was measured again on day. **F)** After 36 days, we collected blood by cardiac puncture. The serum was separated by centrifugation of the samples at 1600 rpm for 20 min to evaluate the serum insulin levels. Values represent the mean±SEM from three independent experiments. *p<0.05; **p<0.01; ***p<0.001; ****p<0.0001. Differences among initial groups (diabetic or not) were analyzed using Student’s t-test. Differences among the groups were tested

with two-way analysis of variance followed by Tukey-Kramer post hoc test. (GraphPad Prism version 6.0 for Windows, GraphPad Software, La Jolla, CA, USA).

Supplemental figure 2

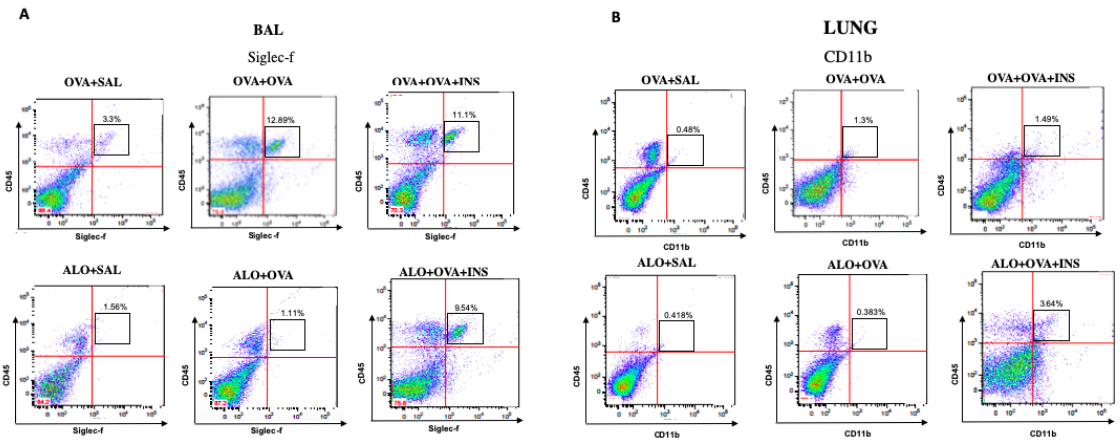


**Supplementary Figure 2. Profile analysis of recruited eosinophils cells.** Acquisitions were performed using an FACSCanto flow cytometer, and data were analyzed using FlowJo software (Tree Star, Inc., USA). We analyzed 20,000 events. Representative dot plots showing the gating strategy used to identify **A)** Siglec-f in BAL and **B)** CD11b in lung.

Supplemental figure 3

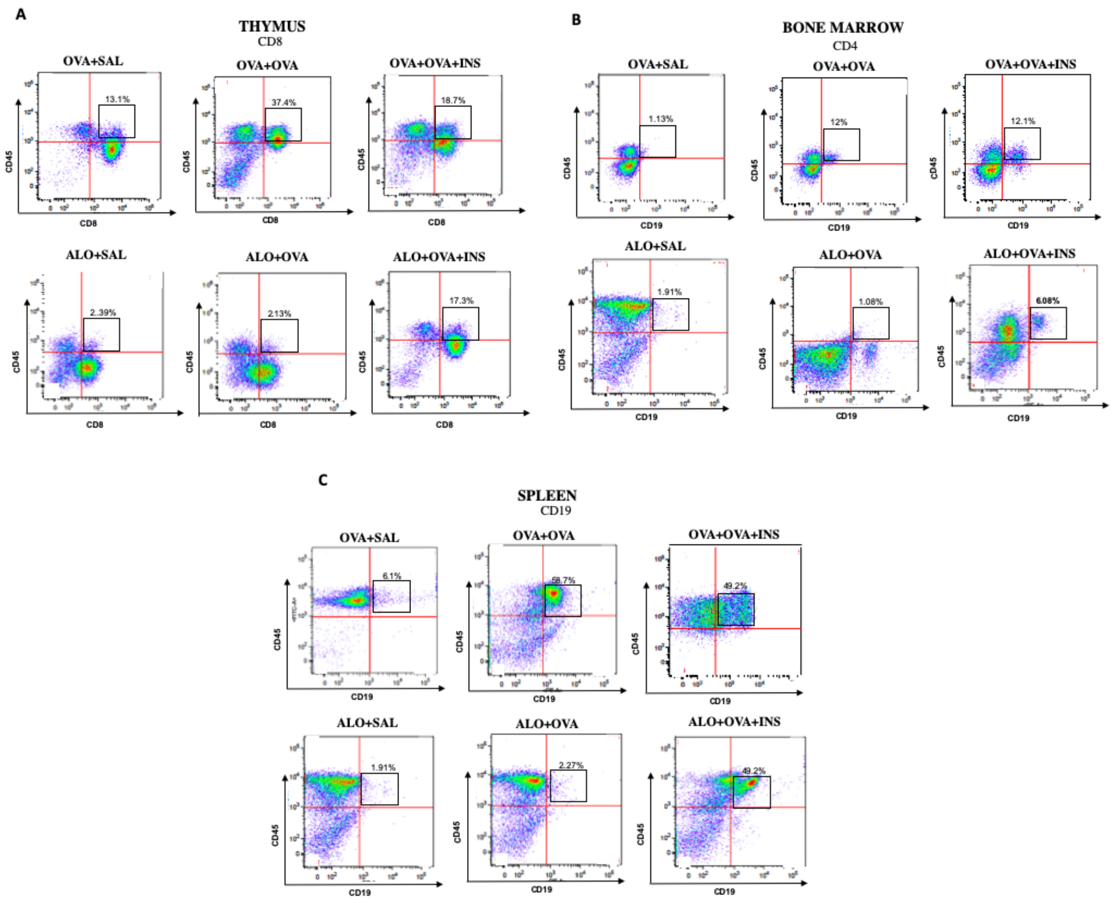


**Supplementary Figure 3. Profile analysis of recruited lymphocytes cells.** Acquisitions were performed using an FACSCanto flow cytometer, and data were analyzed using FlowJo software (Tree Star, Inc., USA). We analyzed 20,000 events. Representative dot plots showing the gating strategy used to identify **A)** CD8 in Thymus and **B)** CD4 in bone marrow **C)** CD19 in spleen.

## Supplemental Table

| LUNG | | | | | | |
|---|---|---|---|---|---|---|
| | OVA+SAL | OVA+OVA | OVA+OVA+INS | ALO+SAL | ALO+OVA | ALO+OVA+INS |
| Total Cells $10^6$/mL | 5.9±0.9 | 52.8±1.5 | 34.3±1.1 | 4.5 ±0.6* | 6.5±0.6** | 30.4±1.5*** |
| SPLEEN | | | | | | |
| | OVA+SAL | OVA+OVA | OVA+OVA+INS | ALO+SAL | ALO+OVA | ALO+OVA+INS |
| Total Cells $10^6$/mL | 143.9±13.9 | 254.7±2.4 | 262.6±7.1 | 18.6±2.1* | 40.2±3.7** | 176.6±2.2*** |
| THYMMUS | | | | | | |
| | OVA+SAL | OVA+OVA | OVA+OVA+INS | ALO+SAL | ALO+OVA | ALO+OVA+INS |
| Total Cells $10^6$/mL | 17.6±1.1 | 48.8±3.3 | 37.4±1.0 | 4.4±1.4* | 7.9±0.9** | 53.8±10.5*** |
| BONE MARROW | | | | | | |
| | OVA+SAL | OVA+OVA | OVA+OVA+INS | ALO+SAL | ALO+OVA | ALO+OVA+INS |
| Total Cells $10^7$/mL | 3.62±0.12 | 3.31±0.23 | 3.3±0.18 | 1.61±0.35* | 1.51±0.14** | 3.10±0.18*** |

**Supplementary Table 1. Total cell values in Lung, Spleen, Thymus and Bone Marrow**. Non-diabetic SAL challenged (OVA+SAL); non-diabetic OVA challenged (OVA+OVA); non-diabetic OVA challenged and treated with insulin (OVA+OVA+INS); diabetic SAL challenged (ALO+OVA); diabetic OVA challenged (ALO+OVA); diabetic OVA challenged and treated with insulin (ALO+OVA+INS). Values represent the mean number of leukocytes ± SEM. (4 animals per group for lung. thymus. and spleen). (6 animals per group – boné marrow). * p<OVA + SAL vs ALO + SAL. ** p <OVA + OVA vs ALO + OVA. *** p <ALO + OVA vs ALO + OVA + INS. Differences among the groups were tested with two-way analysis of variance followed by Tukey-Kramer post hoc test. (GraphPad Prism version 6.0 for Windows, GraphPad Software, La Jolla, CA, USA). *p<0.05; **p<0.01; ***p<0.001

**Supplemental references**


1. Ferreira, S.S., Nunes, F.P.B, Casagrande, F.B., Martins, J.O. Insulin modulates cytokine release, collagen and mucus secretion in lung remodeling of allergic diabetic mice. Frontiers in Immunology (2017) 8:633 doi.org/10.3389/fimmu.2017.00633

2. Spiller, F; Carlos, D; Souto, F.O; de Freitas, A; Soares, F.S; Vieira, S. M; Paula, F. J; Alves-Filho, J. C; Cunha, F. Q. α1-Acid Glycoprotein decreases neutrophil migration and increases susceptibility to sepsis in diabetic mice. Diabetes (2012) 61: 1584. doi:10.2337/db11-0825

3. Stumm C.L., Wettlauer, S.H., Jancar, S., Peters-Golden, M., Airway remodeling in murine asthma correlates with a defect in $PGE_2$ synthesis by lung fibroblasts. Am J Physiol Lung Cell Mol Physiol (2011) 10.1152. doi:10.1152/ajplung.00158.2011

4. Di Petta, A.; Greco, K. V.; Castro, E.O.; Lopes, F.D.T.Q.S.; Martins, M.A.; Capelozzi, V.L. Insulin modulates inflammatory and repair responses to elastase-induced emphysema in diabetic rats. Int J Exp Path. (2011) 92:392-9. doi:10.1111/j.1365-2613.2011.00787.x
